# Development and validation of a prediction model for the prognosis of renal cell carcinoma with liver metastases: a population-based cohort study

**DOI:** 10.3389/fmed.2024.1464589

**Published:** 2024-12-03

**Authors:** Fei Wang, Pan Wang, Xihao Wang, Hengming Lu, Yuchun Han, Lianqu Wang, Zhihui Li

**Affiliations:** ^1^Department of Reproductive Medicine, Central Hospital of Zhumadian, Henan, China; ^2^Department of Urology and Male Reproductive Health, Maternal and Child Health Hospital, Luoyang, China; ^3^Department of Urology, The First Affiliated Hospital of Henan University, Kaifeng, China; ^4^Department of Gastroenterology, Central Hospital of Zhumadian, Henan, China; ^5^Department of Urology, Women and Children's Hospital, Central Hospital of Zhumadian, Henan, China

**Keywords:** renal cell carcinoma, liver metastases, nomogram, prognosis, SEER

## Abstract

**Background:**

Current studies on the establishment of prognostic model for renal cell carcinoma (RCC) with liver metastases (LM) were scarce. This study aimed to develop nomograms to predict the prognosis of RCC with LM.

**Methods:**

Patients diagnosed with RCC between 2010 and 2021 from the Surveillance, Epidemiology, and End Results (SEER) database were selected. The eXtreme Gradient Boosting (XGBoost) and Random Forest (RF) machine learning algorithms were used to screen for the most influential factors affecting prognosis, and the Venn diagram method was employed for further refinement. Subsequently, a nomogram related to brain metastases was constructed. The performance of the nomograms was evaluated through receiver operating characteristics (ROC) curves, calibration plots, C-index, time-dependent C-index, and decision curve analysis (DCA). Kaplan–Meier (K-M) survival curves were used to provide additional verification of the clinical efficacy of the nomogram.

**Results:**

This research comprised 2,395 RCC patients with LM. The Venn diagram demonstrated that age, histological type, grade, AJCC T stage, AJCC N stage, surgery, chemotherapy, marital status, and lung metastasis were highly relevant variables to patients with LM. The AUC, C-index, calibration curves, and DCA curves showed excellent performance of the nomogram. Additionally, the prognostic nomogram accurately classified RCC with LM patients into low- and high-risk groups for mortality.

**Conclusion:**

This study developed a novel nomogram to predict the prognostic factors of RCC with LM, providing a valuable reference for making accurate clinical decisions.

## 1 Introduction

Renal cell carcinoma (RCC) is one of the three major urinary system tumors, accounts for 2 to 3% of global cancer diagnoses and deaths (1). Nevertheless, as a highly malignant tumor, the clinical manifestations of RCC are asymptomatic in the early stages of the disease, and approximately 20 to 30% of patients with RCC have developed distant metastases at the time of their initial diagnosis (2). The 5-year survival rate of metastatic RCC is only about 10%, with a median survival time of less than 10 months (3). In addition, the research has also reported that 90% of tumor related deaths attributed to tumor metastases, instead of the primary tumor (4).

In terms of treatment, although traditional chemotherapeutic drugs are generally ineffective for metastatic RCC (mRCC) patients, systemic therapy has remained the mainstay of treatment for a long time (5). Prior to 2005, several immunotherapeutic agents, for instance interferon-alpha and interleukin-2, were limited therapeutic strategies can be obtained for mRCC, but having shortcomings of low pharmaceutical response rates and noticeable associated toxicity (6, 7). Encouragingly, over the past two decades, targeted therapy and immune checkpoint inhibitors have become new treatment standards for mRCC patients and improve survival rates in individuals with mRCC to some extent (8).

Liver metastases (LM), as one of the commonest metastatic sites of RCC, account for about one-fifth of distant metastases of RCC (9). Previous study has shown that median survival of RCC with LM is 17.6 months, which is inferior to RCC with lung metastases (25.1 months) and RCC with bone metastases (19.4 months) (10). Worse still, RCC has less than 1% complete responses to targeted therapy, and the responses are lower than those observed in cytokine therapies (11). In addition, the objective response rate of LM to systemic immunochemotherapy is only approximately 15% (12). Thus, LM is a significant poor prognostic factor that is worth taking seriously, we need to detect it early and take effective treatment measures to improve survival outcomes of patients.

Nomogram, as a tool commonly utilized in medical research, is usually used to evaluate the incidence or prognosis of a certain disease. It integrates several relevant indicators and builds a graphical prediction tool to provide a single numerical probability of clinical events in an intuitive and simple way to meet the needs of integrated biological and clinical models (13, 14). Nomogram has been widely used in different disease models to assess the risk of a certain disease for a specific individual, providing highly accurate and individualized evidence-based medicine basis, and easy for clinicians to apply and develop more reasonable and standardized diagnosis and treatment plans for patients (15).

Currently, there are no comprehensive reports in a large population to predict prognosis for liver metastatic RCC patients. Therefore, we attempted to establish and validate a nomogram model on prognostic factor for RCC with LM patients based on the Surveillance, Epidemiology, and End Results (SEER) database, which provided individualized guidance for clinicians to develop treatment and follow-up strategies.

## 2 Materials and methods

### 2.1 Data acquisition and data extraction

This study was conducted as a retrospective cohort study using the SEER database.^1^ The SEER database, established by the National Cancer Institute (NCI), is a public cancer statistics resource that provides a comprehensive range of clinical and pathological data, including information on diagnoses, treatments, and survival outcomes for various tumors (16). All data for this study were downloaded from the SEER database [SEER 17 Regs Custom Data, Nov 2023 Sub] using SEER*Stat 8.4.3 software. Since the patient information in the SEER database was anonymous and publicly accessible, ethics committee approval was not required for our study.

**Figure 1:**
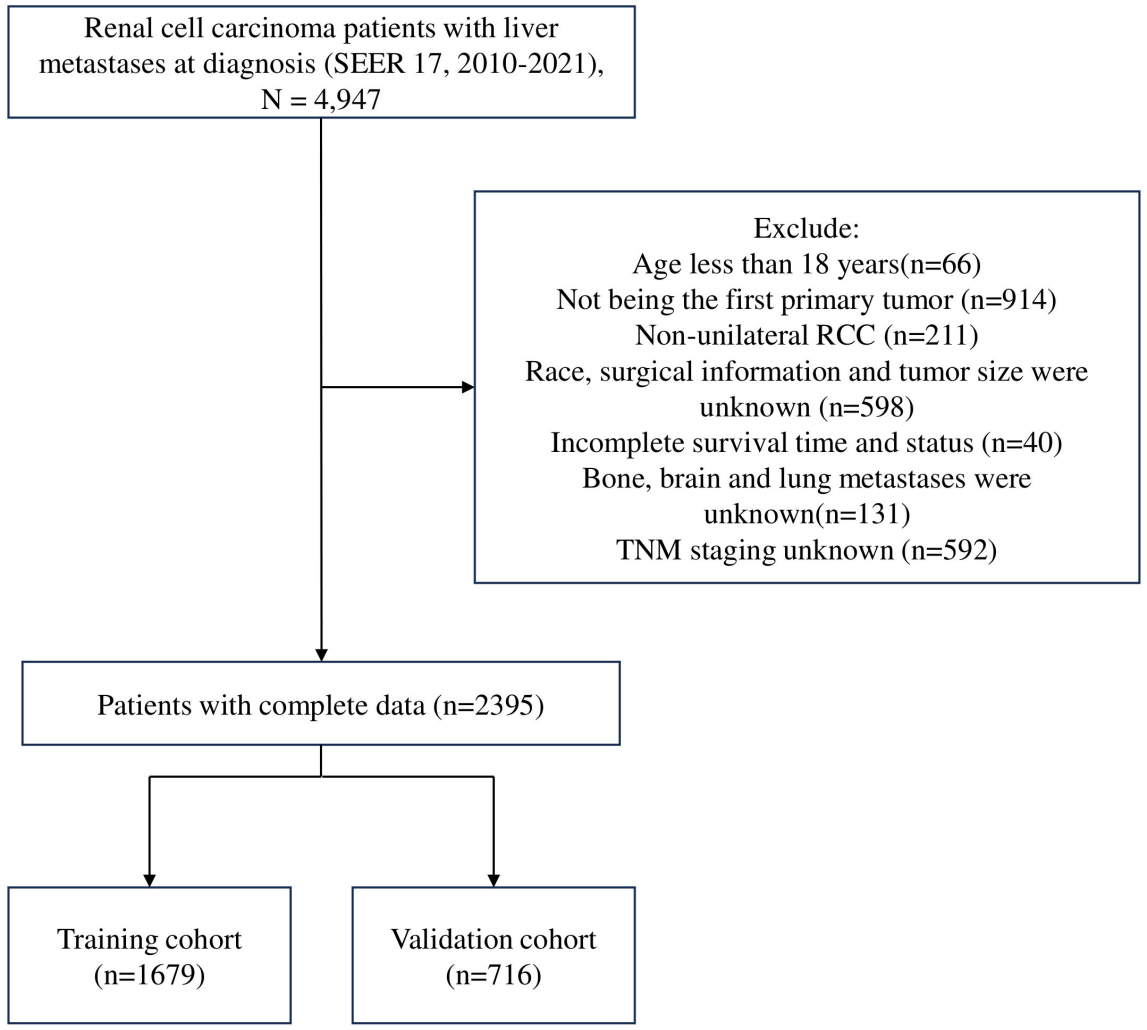
Flowchart of the renal cell carcinoma with liver metastases patient’s selection.

In our study, individuals with LM from RCC diagnosed between 2010 and 2021 were selected based on specific inclusion and exclusion criteria. The inclusion criteria were: (1) RCC confirmed by Site Recode ICD-O-3/WHO 2008 (C64.9, Kidney); (2) diagnosis between 2010 and 2021; and (3) development of LM at the initial diagnosis. The exclusion criteria were: (1) age less than 18 years; (2) presence of two or more primary cancers; (3) non-unilateral RCC; (4) unknown surgical information, survival status, or organs metastases status; (5) unknown exact tumor size; (6) unknown T, N, or M stage; (7) RCC diagnosed only by autopsy or death certificate; and other unspecified criteria. The specific screening process is illustrated in Figure 1.

### 2.2 Variable extraction and cohort identification

The following continuous and categorical data were extracted according to the codes in the SEER database: age at diagnosis (<40 years, 40–49 years, 50–59 years, 60–69 years, 70–79 years, 80 + years), sex (male or female), race (white, black, and other races), histological subtype [clear cell renal cell carcinoma (ccRCC), papillary renal cell carcinoma (pRCC), chromophobe renal cell carcinoma (chrRCC) and others], laterality (left or right), grade [well-differentiated (I), moderately differentiated (II), poorly differentiated (III), undifferentiated (IV) and unknown], surgery [non-surgical group (code 0)], local tumor excision (code 10–27; includes cryosurgery, thermal ablation, laser excision), and partial nephrectomy (code 30) and radical nephrectomy (code 40–80), therapy (radiotherapy and chemotherapy), tumor size (divide into <94 mm and ≥ 94 mm based on the median), marital status (married, unmarried/others), distant metastases (bone, brain, or lung metastases), survival status, and survival time. Additionally, according to the AJCC TNM system, the T-stage is divided into T1–T4 and the N-stage into N0–N1.

### 2.3 Variable selection

All eligible patients were randomly divided into training and validation cohorts at a ratio of 7:3. In contrast to traditional methods for screening variable importance, this study utilizes a model coefficient-dependent approach to analyze the significance of variables in the training cohort. Specifically, machine learning techniques are employed to rank the features of the included variables, thereby extracting the most important ones. Two machine learning methods, eXtreme Gradient Boosting (XGBoost) and Random Forest (RF), were used to perform the variable screening operations (17–19). Subsequently, Venn diagrams were performed to select the common variables filtered by both RF and XGBoost models, providing a visual demonstration of the consistency between the two models. This method helped identify variables that were considered important by different machine learning methods, thereby enhancing the reliability and interpretability of the results.

### 2.4 Nomogram construction and validation

Variables identified by the Venn diagrams were treated as independent prognostic risk factors for patients with LM. A nomogram was then constructed to predict 1-, 2-, and 3-year OS for RCC patients with LM. We employed the C-index, area under the curve (AUC), time-dependent receiver operating characteristic (ROC) curves, and calibration curves to verify the accuracy of the training and validation cohorts. Additionally, decision curve analysis (DCA) was utilized to evaluate the clinical efficacy of the model. Patients were divided into low-risk and high-risk groups based on the total scores from the nomogram. Kaplan–Meier curves and log-rank tests were employed to compare the survival rates between the different groups.

### 2.5 Statistical analysis

Pearson’s chi-square analysis was used to evaluate categorical variables. A novel nomogram was developed based on prognostic factors. The log-rank test and Kaplan–Meier (K-M) curves were used to analyze the survival differences among patients. All statistical analyses were performed using R software (version 4.2.3).^2^ Statistical significance was defined as a two-tailed *p*-value of less than 0.05.

## 3 Results

### 3.1 Clinical characteristics of eligible patients

According to our rigorous screening, the entire cohort comprised 2,395 RCC patients with LM from SEER database. 1,679 cases served as training cohort, and the remaining 716 patients were internal validation cohort. Table 1 presents baseline clinical features and treatment regimens of LM patients. The number of patients under 40 years old was 62 (2.6%), 40–49 years old was 193 (8.1%), 50–59 years old was 564 (23.5%), 60–69 years old was 776 (32.4%), 70–79 years old was 504 (21.0%), and over 79 years old was 296 (12.4%). The study found that among all patients, 1,531 (63.9%) were male, 1,890 (78.9%) were white, and 1,079 (45.1%) were married. A total of 839 (35.0%) patients were diagnosed with ccRCC, and there were 1,299 (54.2%) patients with tumors on the left side. 22 (0.9%), 127 (5.3%), 341 (14.2%), 306 (12.8%) and 1,599 (66.8%) patients whose Fuhrman grade were I, II, III, IV and unknown, respectively. Tumor stages were T1 (445, 18.6%), T2 (564, 23.5%), T3 (867, 36.2%), and T4 (519, 21.7%). Among the patients, 1,289 (53.8%) had stage N0, and 1,106 (46.2%) had stage N1. 1,807 (75.4%) patients did not undergo surgical treatment, while 559 (23.3%) underwent radical nephrectomy. The total number of patients who received radiotherapy was 480 (20.0%), and 1,085 (45.3%) received chemotherapy. 1,207 (50.4%) patients had tumors smaller than 94 mm. A total of 892 (37.2%) patients had bone metastases, 251 (10.5%) had brain metastases, and 1,485 (62.0%) had lung metastases. Except for pathological grade and chemotherapy, no significant differences were observed in other variables between the training and validation cohorts.

**Table 1:** Demographic and clinicopathological characteristics for renal cell carcinoma with liver metastases patients.

Characteristics	Whole population (*n* = 2,395)	Training cohort (*n* = 1,679)	Validation cohort (*n* = 716)	*p*
Age (years)				0.430
<40	62 (2.6%)	43 (2.6%)	19 (2.7%)	
40–49	193 (8.1%)	146 (8.7%)	47 (6.6%)	
50–59	564 (23.5%)	396 (23.6%)	168 (23.5%)	
60–69	776 (32.4%)	528 (31.5%)	248 (34.6%)	
70–79	504 (21.0%)	360 (21.4%)	144 (20.1%)	
80+	296 (12.4%)	206 (12.3%)	90 (12.6%)	
Sex				0.802
Male	1,531 (63.9%)	1,076 (64.1%)	455 (63.6%)	
Female	864 (36.1%)	603 (35.9%)	261 (36.5%)	
Race				0.364
White	1890 (78.9%)	1,313 (78.2%)	577 (80.6%)	
Black	308 (12.9%)	226 (13.5%)	82 (11.4%)	
Other	197 (8.2%)	140 (8.3%)	57 (8.0%)	
Marital status				0.759
Married	1,079 (45.1%)	753 (44.8%)	326 (45.5%)	
Unmarried/Unknown	1,316 (54.9%)	926 (55.2%)	390 (54.5%)	
Histologic Type				0.998
ccRCC	839 (35.0%)	590 (35.1%)	249 (34.8%)	
pRCC	86(3.6%)	60 (3.6%)	26 (3.6%)	
chrRCC	27 (1.1%)	19 (1.1%)	8 (1.1%)	
Other	1,443 (60.3%)	1,010 (60.2%)	433 (60.5%)	
Grade				0.020
I	22 (0.9%)	17 (1.0%)	5 (0.7%)	
II	127 (5.3%)	87 (5.2%)	40 (5.6%)	
III	341 (14.2%)	248 (14.8%)	93 (13.0%)	
IV	306 (12.8%)	236 (14.1%)	70 (9.8%)	
Unknown	1,599 (66.8%)	1,091 (64.9%)	508 (70.9%)	
AJCC T stage				0.346
T1	445 (18.6%)	300 (17.9%)	145 (20.3%)	
T2	564 (23.5%)	394 (23.5%)	170 (23.7%)	
T3	867 (36.2%)	625 (37.2%)	242 (33.8%)	
T4	519 (21.7%)	360 (21.4%)	159 (22.2%)	
AJCC N stage				0.677
N0	1,289 (53.8%)	899 (53.5%)	390 (54.5%)	
N1	1,106 (46.2%)	780 (46.5%)	326 (45.5%)	
Laterality				0.764
Left	1,299 (54.2%)	914 (54.4%)	385 (53.8%)	
Right	1,096 (45.8%)	765 (45.6%)	331 (46.2%)	
Surgery				0.806
No	1807 (75.4%)	1,259 (75.0%)	548 (76.5%)	
Local excision	11 (0.5%)	7 (0.4%)	4 (0.6%)	
Partial Nephrectomy	18 (0.8%)	13 (0.8%)	5 (0.7%)	
Radical Nephrectomy	559 (23.3%)	400 (23.8%)	159 (22.2%)	
Radiation				0.540
No	1915 (80.0%)	1,337 (79.6%)	578 (80.7%)	
Yes	480 (20.0%)	342 (20.4%)	138 (19.3%)	
Chemotherapy				0.004
No	1,310 (54.7%)	886 (52.8%)	424 (59.2%)	
Yes	1,085 (45.3%)	793 (47.2%)	292 (40.8%)	
Size				0.157
<94 (mm)	1,207 (50.4%)	862 (51.3%)	345 (48.2%)	
≥94 (mm)	1,188 (49.6%)	817 (48.7%)	371 (51.8%)	
Bone metastases				1.000
No	1,503 (62.8%)	1,054 (62.8%)	449 (62.7%)	
Yes	892 (37.2%)	625 (37.2%)	267 (37.3%)	
Brain metastases				0.666
No	2,144 (89.5%)	1,506 (89.7%)	638 (89.1%)	
Yes	251 (10.5%)	173 (10.3%)	78 (10.9%)	
Lung metastases				0.786
No	910 (38.0%)	635 (37.8%)	275 (38.4%)	
Yes	1,485 (62.0%)	1,044 (62.2%)	441 (61.6%)	

ccRCC, clear cell renal cell carcinoma; pRCC, papillary renal cell carcinoma; chrRCC, chromophobe renal cell carcinoma.

### 3.2 Feature selection for model development

**Figure 2:**
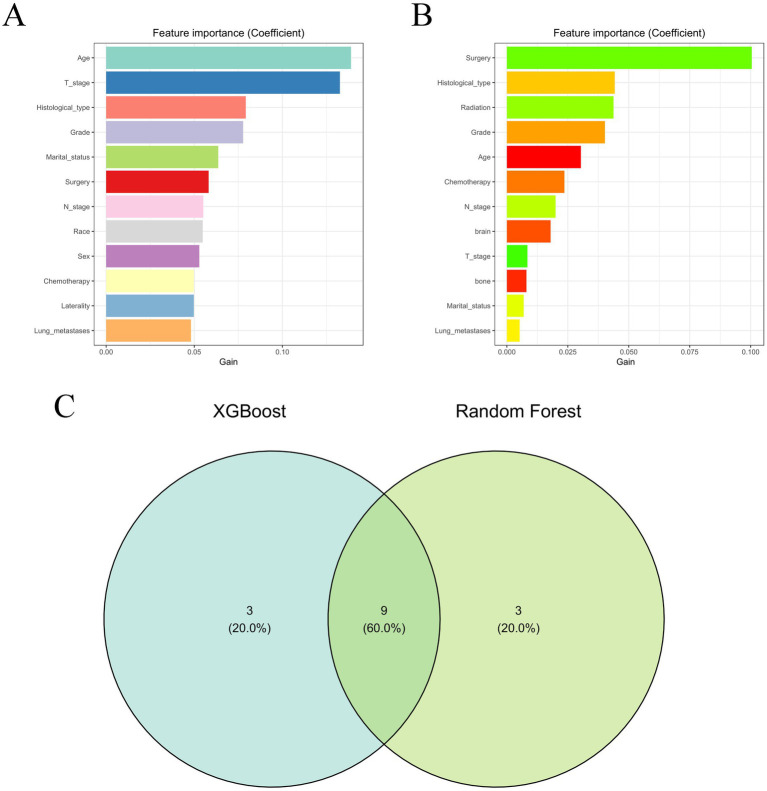
The results of XGBoost **(A)** and RF **(B)** machine learning algorithms filter the top 13 important variables. The results are expressed by coefficient value. **(C)** Venn analysis of the results of the above two machine algorithms.

The optimization of feature variables was conducted through the application of machine learning algorithms, namely XGBoost (Figure 2A) and RF (Figure 2B). Each algorithm separately identified the top 12 most important feature variables for their respective models. Subsequently, through comprehensive analysis using Venn diagrams, 10 variables (age, histological type, T stage, N stage, surgery, chemotherapy, lung metastases, and marital status) were identified for the construction of the prognostic model (Figure 2C).

### 3.3 Prognostic nomogram model construction and validation

**Figure 3:**
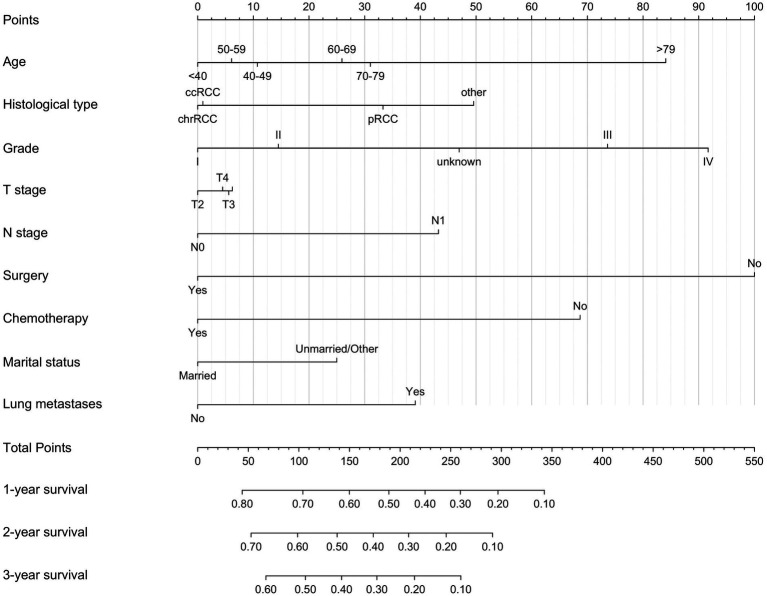
Nomogram for predicting 1-, 2-, and 3-year OS for renal cell carcinoma with liver metastases patients in the training cohort.

**Figure 4:**
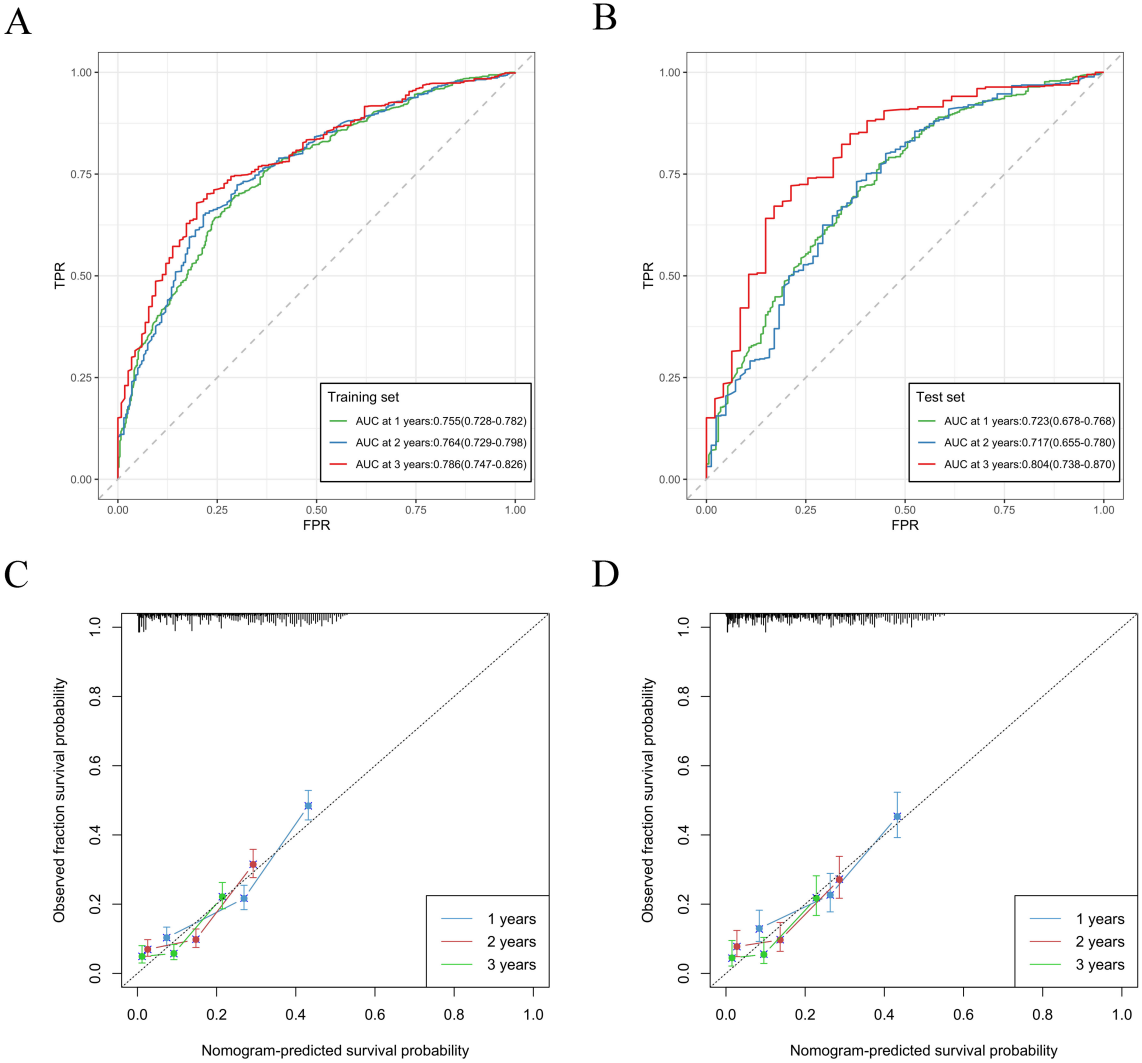
Nomogram ROC curves to predict 1-, 2-, and 3-year OS in the training cohort **(A)** and in the validation cohort **(B)**. Nomogram calibration curves to predict 1-, 2-, and 3-year OS in the training cohort **(C)** and in the validation cohort **(D)**.

**Figure 5:**
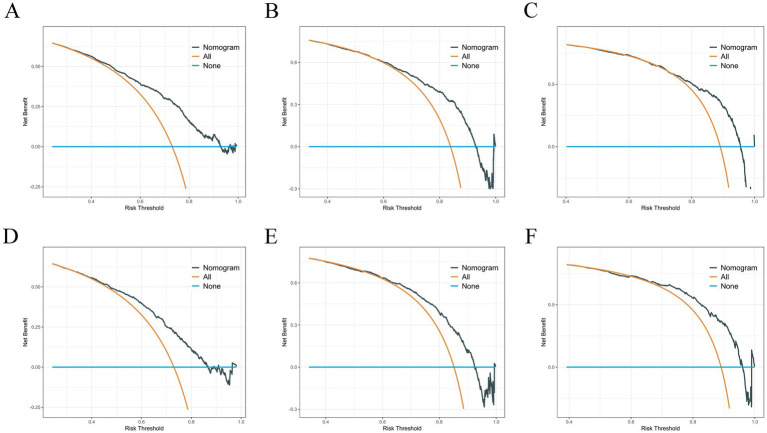
**(A–C)** DCA analysis predicting 1-, 2-, and 3-year OS in the training cohort. **(D–F)** DCA analysis predicting 1-, 2-, and 3-year OS in the validation cohort.

Based on variables that Venn diagrams identified in the training cohort, we constructed a predictive nomogram model of RCC with LM patients (Figure 3). The C-index were 0.715(0.701–0.729) and 0.702(0.678–0.726) in the training and validation cohorts, which indicated that the model had good discrimination. The AUC values were 0.755(0.728–0.782), 0.764(0.729–0.798) and 0.786(0.747–0.826) regarding nomogram predicting 1-, 2-, and 3-year OS in the training cohort (Figure 4A), and the AUC values were 0.723(0.678–0.768), 0.717(0.655–0.780) and 0.804(0.738–0.870) regarding nomogram predicting 1-, 2-, and 3-year OS in the validation cohort (Figure 4B). The calibration curves of the nomogram showed high consistency between the predicted and actual probabilities of OS in the training cohort (Figure 4C) and the validation cohort (Figure 4D). Furthermore, DCA curves demonstrated that nomogram could better predict OS and had high clinical practical value (Figure 5).

### 3.4 Risk stratification system

**Figure 6:**
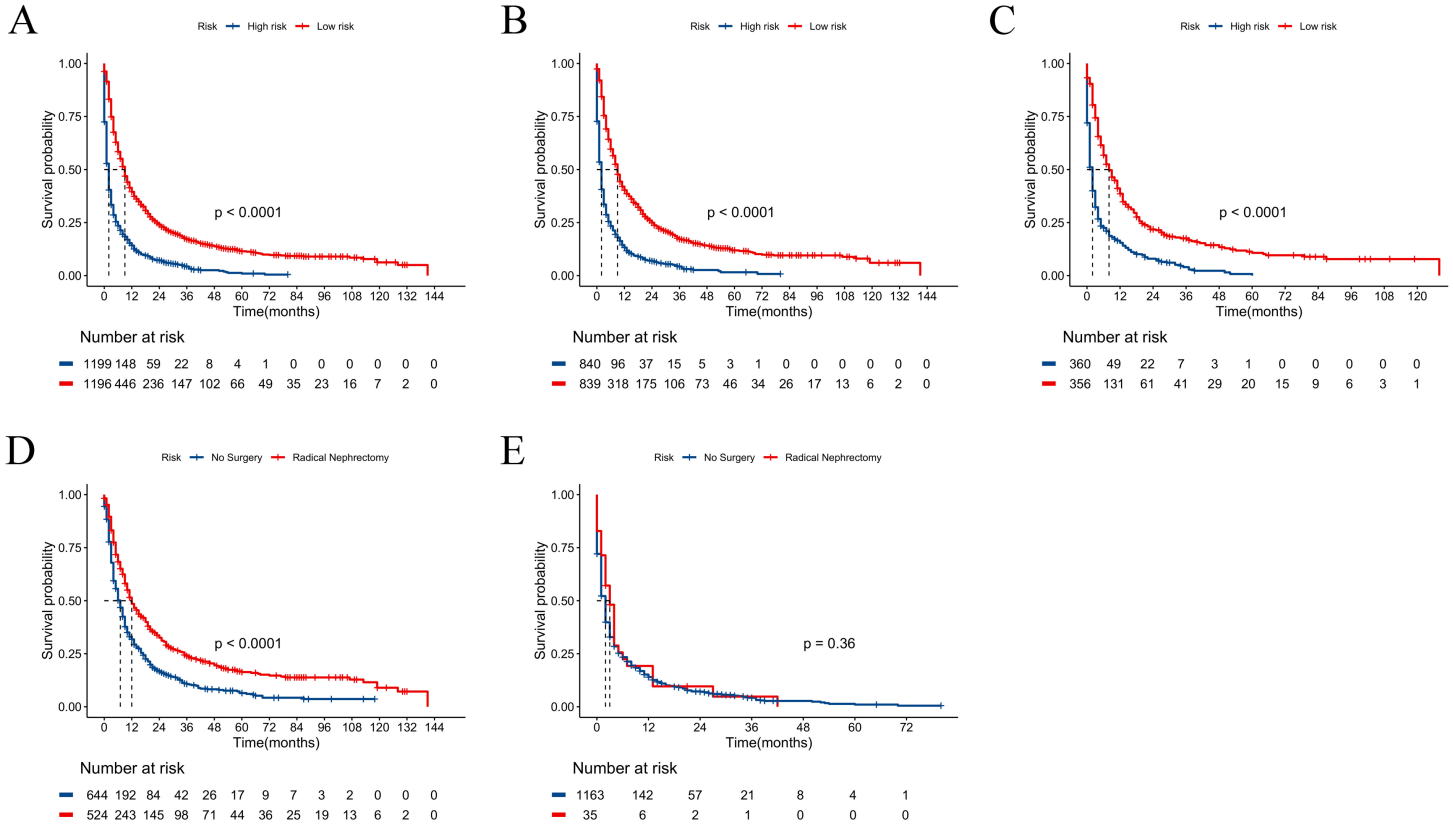
Kaplan–Meier curves for predicting OS of patients in low-risk and high-risk groups. **(A)** For all cohort; **(B)** For training cohort; **(C)** For validation cohort. Kaplan–Meier curves predicting OS of patients receiving radical nephrectomy versus not receiving surgery. **(D)** In low-risk group; **(E)** In high-risk group.

Utilizing the patients’ total scores from the nomogram, we developed a risk stratification system. The patients were divided into low-risk and high-risk groups by calculating the risk scores using all independent prognostic factors, with the median used as the cut-off value. The Kaplan–Meier survival analysis indicated that patients in the low-risk group had a significantly better prognosis than those in the high-risk group (Figures 6A–C). Additionally, we compared the influence of receiving radical nephrectomy versus not receiving surgery on the survival probability of patients in the low-risk and high-risk groups. In the low-risk group, patients receiving radical nephrectomy had a better survival probability than those not receiving surgery (Figure 6D). In the high-risk group, no significant difference was found in survival probability between patients receiving radical nephrectomy and those not receiving surgery (Figure 6E), possibly due to the small sample size of patients.

## 4 Discussion

As one of the three major malignant tumors of the urinary system, RCC is characterized by high tumor heterogeneity and high recurrence rate, and accounts for 90% of adult renal tumors (20). RCC is resistant to traditional radiotherapy and chemotherapy. The current therapeutic options of advanced RCC includes chemotherapy, targeted therapy, and immunotherapy (21). The liver is one of the most common metastatic sites for various solid tumors, especially gastrointestinal tumors, such as colorectal cancer, gastric cancer, and pancreatic cancer (22–24). Approximately 23.6% of metastatic sites in newly diagnostic RCC patients are in the liver, and they experienced a poor OS (25). ML is a robust computational technique proficient in managing complex and voluminous datasets. It can handle highly variable data, extracting nonlinear and seemingly unrelated factors that traditional methods may overlook, thereby achieving more precise feature selection (26). In this paper, we utilized XGBoost and RF algorithms, employed Venn diagrams to identify 9 key variables influencing the prognosis of patients with LM, and constructed a prognostic model. As far as we know, this research was the first larger scale population-based study that described prognostic factors of RCC with LM patients. Further, a practical predictive and prognostic model for RCC with LM was constructed to help clinicians formulate personalized treatment strategies.

Our study found that older people had a worse prognosis of LM patients than younger patients. It might be related to the deterioration of physical ability and the decrease of immune system function of the elderly (27). Moreover, it was relatively consistent with previous research results, which age was an independent risk factor for LM (23, 28). Currently, there was no consensus on the relationship between RCC histological subtype and tumor liver metastasis. In this study, concerning histological subtype, RCC patients with ccRCC and chrRCC with LM patients were more likely to suffer a better OS. It was similar to previous research results (11). However, Dong S et.al found the highest risk histological subtype for RCC with bone metastasis was ccRCC and renal cell carcinoma (29). Jiang L et.al indicated that sarcomatoid RCC had a high incidence of lung metastases, and collecting duct carcinoma with lung metastases had a worse prognosis (30).

Another finding suggested that undifferentiated pathological types of RCC were more prone to LM, it was relatively consistent with previous research (29). The reason why poorly differentiated tumors were prone to distant metastasis might be that higher grade meant more biological aggressiveness (31). In our study, T stage and N stage were independent risk factors for the development of RCC with LM. As essential parts of TNM staging system, T stage indicated the tumor size, depth, and location of tumor infiltration, and N stage indicated the site and number of lymphatic metastases, higher T stage and N stage increased the possibility of distant metastasis (30, 32). N1stage tumors of LM had a worse prognosis than N0 tumors in N stage. It was reported that N stage had significant contribution to prognosis of synchronous lung metastasis in renal cell carcinoma (33).

Notably, the role of surgical intervention in managing mRCC remains debated, particularly in the context of the current era’s focus on targeted therapies and immunotherapy (34, 35). According to a large clinical trial, researchers observed that in certain patients with intermediate-risk or poor-risk mRCC, Sunitinib monotherapy was non-inferior to Sunitinib combined with CN (36). However, in a retrospective study utilizing the REMARCC (Registry of Metastatic RCC) database, Meagher et al. noted that patients who underwent CN and received systemic therapy had improved prognosis, with those treated with immunotherapy showing better outcomes compared to those receiving therapies targeting specific proteins, such as tyrosine kinase inhibitors (TKIs) (37). Takemura et al. analyzed data from the International mRCC Database Consortium (IMDC) and found that in select mRCC patients receiving frontline immuno-oncology (IO)-based combination therapies, the addition of CN conferred a survival benefit (38). Moreover, in a real-world multi-institutional analysis, Ghatalia et al. demonstrated that mRCC patients who received both CN and systemic therapy experienced greater survival benefits compared to those who underwent systemic therapy alone at earlier stages (39). Interestingly, our study also revealed that RCC patients with LM could lower the risk of LM progression and achieve survival benefits through CN, aligning with existing research.

In clinical practice, healthcare professionals have historically relied on the Memorial Sloan Kettering Cancer Center (MSKCC) and IMDC criteria for risk stratification of patients with mRCC, utilizing these criteria to identify populations that may benefit from CN (40). Nonetheless, these two scoring systems have their own limitations in clinical application, as they fail to account for the complexity of the primary tumor, specific pathologic characteristics, and the clinical skills of the surgeon. A novel scoring system developed by Marchioni et al. that integrated tumor and patient characteristics established a robust basis for selecting mRCC patients who might benefit from CN (41). The research revealed that the most significant tumor factors influencing prognosis were metastatic organ location and the presence of more than three metastatic organs, while obesity and physical activity status were the primary contributors to differences in survival (41). Silagy et al. suggested that clinicians should consider tumor factors (such as high metastatic burden, unresectable primary tumors, and rapid disease progression) and the patient’s own health status (such as poor kidney function and complex comorbidities) when performing CN for patients (42). Furthermore, in a study utilizing an international multicenter cohort, Meagher et al. reclassified the ‘M’ stage of the current AJCC TNM system into two groups based on tumor number: M1 (≤3, ‘Oligometastatic’) and M2 (>3, ‘Polymetastatic’)—to reassess the impact of tumor burden on survival outcomes in mRCC patients, and found that this stratification may offer a more rational and accurate approach to guiding management strategies while yielding results comparable to those predicted by the existing Mozer/Heng standards for prognosis (43).

Additionally, patients who had lung metastases were more prone to occur LM, which also resulted in a worse prognosis. It could be due to the presence of metastases to distant organs at first diagnosis of RCC, showing that tumor cells have escaped via vascular system or other modes of metastases, which greatly increased the possibility of LM (44, 45). Dong et al. reported that LM, lung metastases and brain metastases were independent risk factors for RCC with bone metastasis (29). Similarly, Jiang et al. also confirmed that multiple organs metastases obviously increased the incidence of bone metastases for elderly RCC and lead to adverse outcomes (30). Interestingly, we observed that married patients with LM had a better survival outcome compared to unmarried patients. Potential explanations include the higher levels of stress, social isolation, and lack of support that unmarried individuals were likely to experience, which might contribute to lower survival rates for cancer patients. This was consistent with existing research findings (46, 47).

However, some limitations should be considered in this cohort. Firstly, the sample size of this study is limited, as the SEER database only provides data on distant metastases to specific organs (bone, brain, liver, and lung) from 2010 onward. Secondly, the SEER database does not collect detailed information on the treatment of patients with LM from RCC, preventing us from conducting a more in-depth analysis of the prognosis for these patients. Thirdly, key pathological features known to influence prognosis are not available, including the presence of sarcomatoid/rhabdoid components, invasion of perinephric and/or sinus adipose tissue, and histomorphological subtypes (48, 49). Finally, the data in this study are derived from the US population, which may limit their applicability to patients with brain metastases from renal cell carcinoma in other countries or regions. Consequently, additional multicenter clinical studies are required to validate the accuracy of our model.

## 5 Conclusion

In this study, two innovative nomograms were constructed to assess the risk variables associated with LM and predict the prognostic factors associated with OS for liver metastatic RCC patients. Our study might provide clinicians and RCC patients with a practical tool to prevent LM and improve survival rates in individuals who had developed LM, which might enhance their quality of life.

## Data Availability

The original contributions presented in the study are included in the article/Supplementary material, further inquiries can be directed to the corresponding authors.

## Footnotes

^1^ http://seer.cancer.gov/

^2^ https://www.r-project.org/
